# Minimal hepatic encephalopathy is associated with expansion and activation of CD^4+^CD28^−^, Th22 and Tfh and B lymphocytes

**DOI:** 10.1038/s41598-017-05938-1

**Published:** 2017-07-27

**Authors:** Alba Mangas-Losada, Raquel García-García, Amparo Urios, Desamparados Escudero-García, Joan Tosca, Remedios Giner-Durán, Miguel Angel Serra, Carmina Montoliu, Vicente Felipo

**Affiliations:** 1 Fundación Investigación Hospital Clínico. Instituto Investigación Sanitaria-INCLIVA, Valencia, Spain; 2 Laboratory Neurobiology, Centro Investigación Príncipe Felipe, Valencia, Spain; 3 Unidad de Digestivo, Hospital Clínico Valencia, Departamento de Medicina, Universidad Valencia, Valencia, Spain; 4 Servicio de Digestivo, Hospital Arnau de Vilanova, Valencia, Spain; 5 Departamento de Patología, Facultad de Medicina, Universidad de Valencia, Valencia, Spain

## Abstract

Peripheral inflammation acts synergistically with hyperammonemia in inducing neurological alterations in cirrhotic patients with minimal hepatic encephalopathy (MHE). We hypothesized that appearance of MHE would be associated to some specific qualitative change in peripheral inflammation. The aim of this work was to characterize the changes in peripheral inflammation associated to appearance of MHE. We analyzed it by immunophenotyping and cytokine profile analysis, in cirrhotic patients without or with MHE and controls. The main alterations associated specifically with MHE are: 1) increased activation of all subtypes of CD4^+^ T-lymphocytes, with the increased expression of CD69; 2) increased amount of CD4^+^CD28^−^ T lymphocytes, associated with increased levels of CX3CL1 and of IL-15; 3) increased differentiation of CD4^+^ T lymphocytes to Th follicular and Th22; 4) increased activation of B lymphocytes and serum IgG. This study has identified some specific alterations of the immune system associated with appearance of the neurological alterations in MHE patients.

## Introduction

Patients with liver cirrhosis may suffer minimal hepatic encephalopathy (MHE), with attention deficits, psychomotor slowing, mild cognitive impairment and motor in-coordination which impair quality of life and reduces life span. Hyperammonemia and inflammation play synergistic roles in inducing the neurological alterations in MHE^1–5^. In cirrhotic patients, hyperammonemia deteriorates neuropsychological test scores during inflammatory state but not after its resolution^2^ and markers of inflammation are higher in patients with MHE^3^, indicating that inflammation exacerbates the neuropsychological alterations induced by hyperammonemia^2, 3^. The presence of certain levels of hyperammonemia and inflammation are enough to induce mild cognitive impairment, even in the absence of liver cirrhosis^4^. The levels of the inflammatory cytokines IL-6 and IL-18 in serum of cirrhotic patients correlate with the presence of MHE^5^.

There is increasing evidence that many diseases associated with chronic inflammation lead to neurological impairment resulting in different forms of cognitive and motor alterations. Peripheral inflammation may lead to cognitive alterations in different pathological situations such as diabetes, rheumatoid arthritis, obesity or chronic kidney disease^6^. Inflammation and neuroinflammation also contribute to cognitive and motor deficits in post-operative cognitive dysfunction, ageing and in some mental (schizophrenia) and neurodegenerative (Alzheimer’s) diseases^6–15^. There is also an association between the inflammatory marker IL-6 and cognitive decline in old persons^16–18^.

Patients with chronic inflammatory diseases are being treated with anti-TNFα to reduce peripheral inflammation. This treatment improves cognitive function in patients with rheumatoid arthritis or sarcoidosis^19, 20^. These studies suggest that peripheral inflammation may lead to cognitive alterations in different pathological situations.

The role of peripheral inflammation in the induction of the cognitive and motor alterations in MHE has been also recently demonstrated in animal models (rats with portacaval shunts). Dadsetan *et al*.^21, 22^ showed that treatment with anti-TNFα, which does not cross the blood-brain barrier, acting only peripherally, prevents the induction of inflammation, neuroinflammation and cognitive and motor alterations in rats with MHE due to portacaval anastomosis.

All above studies support that peripheral inflammation plays a main role in the induction of MHE. Patients without MHE already show some grade of inflammation, but it is higher in patients with MHE. The enhanced inflammation seems to trigger cognitive impairment and MHE^3, 5^. We hypothesized that, in cirrhotic patients, MHE appearance would be associated to specific qualitative changes in peripheral inflammation. To assess this possibility we characterized changes in the innate and adaptive immune systems in cirrhotic patients with and without MHE.

A main component of the innate system is the monocyte, showing three subsets with distinct phenotype and function: classical CD14^++^CD16^−^, intermediate CD14^++^CD16^+^ and non-classical CD14^+^CD16^++^ monocytes. Intermediate CD14^++^CD16^+^ show higher expression of pro-inflammatory cytokines^23^.

The main components of the adaptive system are T and B lymphocytes. T helper (Th) lymphocytes (CD4^+^ T cells), are central to the majority of adaptive immune responses and may be naive or memory CD4^+^ T cells, generated following an immune response.

Most CD4^+^ lymphocytes are also CD28^+^ and require both T cell receptor (TCR) stimulation and CD28 co-stimulation for T cell activation. However, there is a CD4^+^CD28^−^ subset of T lymphocytes that have an inbuilt pro-inflammatory ability and are increased and catalyze inflammation in several inflammatory disorders such as autoimmunity, atherosclerosis and chronic viral infections^24–26^.

When CD4^+^ T cells recognize an antigen on an antigen presenting cell they become activated. Once activated, they divide and differentiate into one of several subtypes, including Th1, Th2, Th17, Th22, iTreg or Tfh, which secrete different cytokines to facilitate different types of immune responses^27^.

B lymphocytes participate in the adaptive immune system by presenting antigen and secreting antibodies and cytokines^28^. Activation of T or B lymphocytes induces the expression of the early activation marker CD69^29, 30^.

The aim of this work was to characterize changes in peripheral inflammation associated specifically to MHE. To reach this aim we analyzed by immunophenotyping and cytokine profile analysis, changes in cirrhotic patients without or with MHE compared to controls without liver disease in:

- Monocytes subsets: classical CD14^++^CD16^−^, intermediate CD14^++^CD16^+^ and non-classical CD14^+^CD16^++^
- Pro-inflammatory CD4^+^CD28^−^ subset and memory vs naive CD4^+^ T lymphocytes
- Activation of T and B lymphocytes by measuring the early activation marker CD69 and IgG levels
- Differentiation of CD4^+^ T lymphocytes to different subtypes: Th1, Th2, Th17, Th22, iTreg or Tfh
- The profile of serum cytokines: IL-6, IL-10, TNFα, CCL20, CXCL13, IL-15, CX3CR1, TGFβ, IL-21, IL-17, IL-12, IL-18, IL-1β and IL-22

## Results

Patients were classified in 125 without and 62 with MHE according to PHES score. There were no differences between cirrhotic patients without and with MHE in any marker of liver damage: transaminases, MELD, Child Pugh, or in ammonia, sodium TSH or hemoglobin levels (Supplementary Table 1).

**Figure 1:**
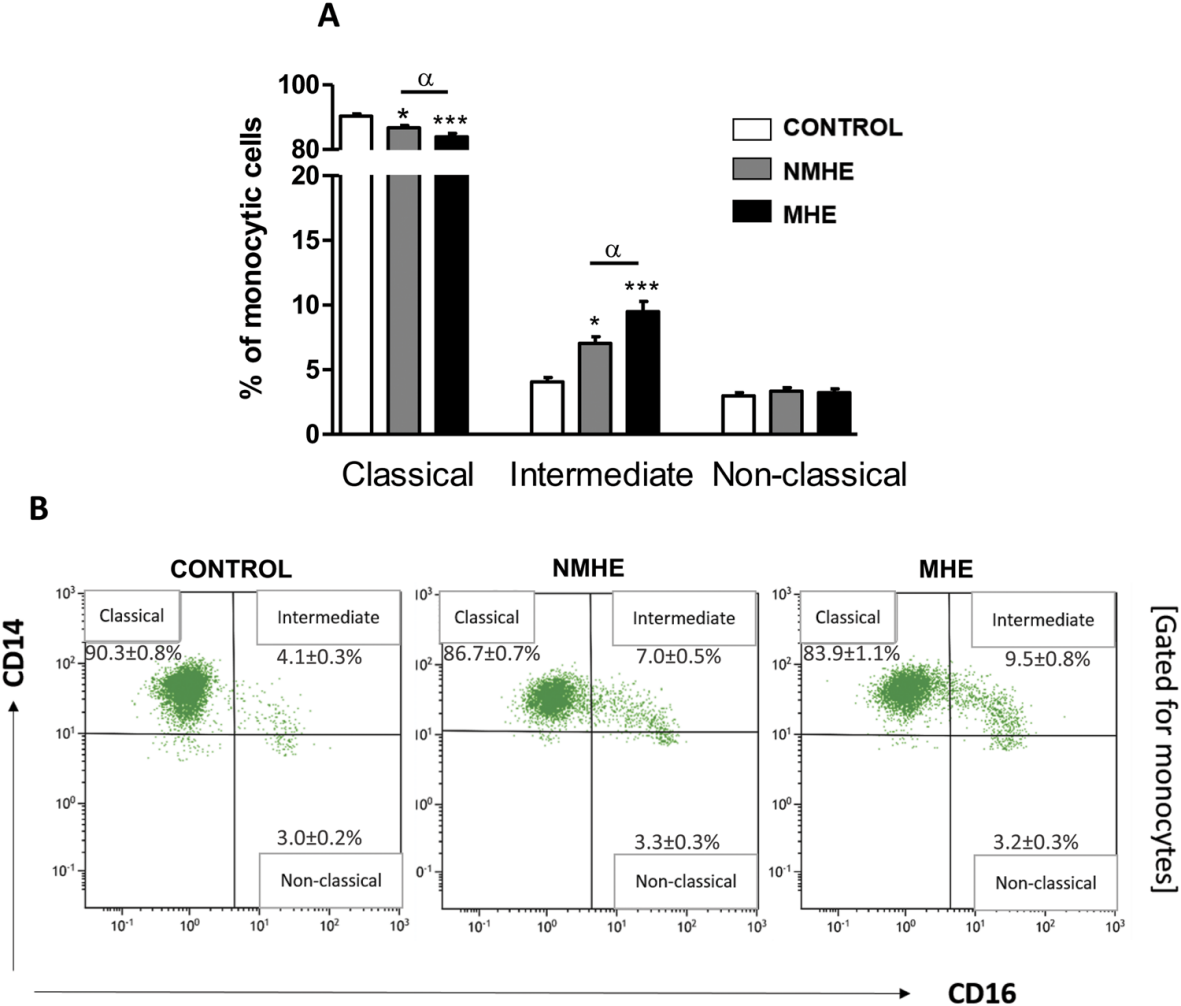
Monocytes populations in peripheral blood. (**A**) Percentage of the three subsets of monocytes over total monocyte cells: classical (CD14^++^CD16^−^), intermediate (CD14^++^CD16^+^) and non-classical (CD14^+^CD16^++^). (**B**) Examples of panels from the cytometer (gated for total monocytes population). Values are the mean ± SEM of the following number of individuals: CONTROL n = 28; without MHE (NMHE) n = 30; with MHE (MHE) n = 40. Values significantly different from controls are indicated by asterisks*. Values significantly different in patients with MHE compared to NMHE are indicated by α. (*/α p < 0.05; ***p < 0.001).

Cirrhotic patients without MHE show increased percentage of intermediate CD14^++^CD16^+^, pro-inflammatory monocytes (7.0 ± 0.5%, p < 0.05) compared to controls (4.1 ± 0.3%). The increase was larger in patients with MHE, who reached 9.5 ± 0.8% (p < 0.001). This was associated with reduced percentage of classical, non-inflammatory CD14^++^CD16^−^ monocytes, that represented 90.3 ± 0.8% in controls, 86.7 ± 0.7% (p < 0.05) in patients without MHE and 83.9 ± 1.1% (p < 0.001) in patients with MHE (Fig. 1).

**Figure 2:**
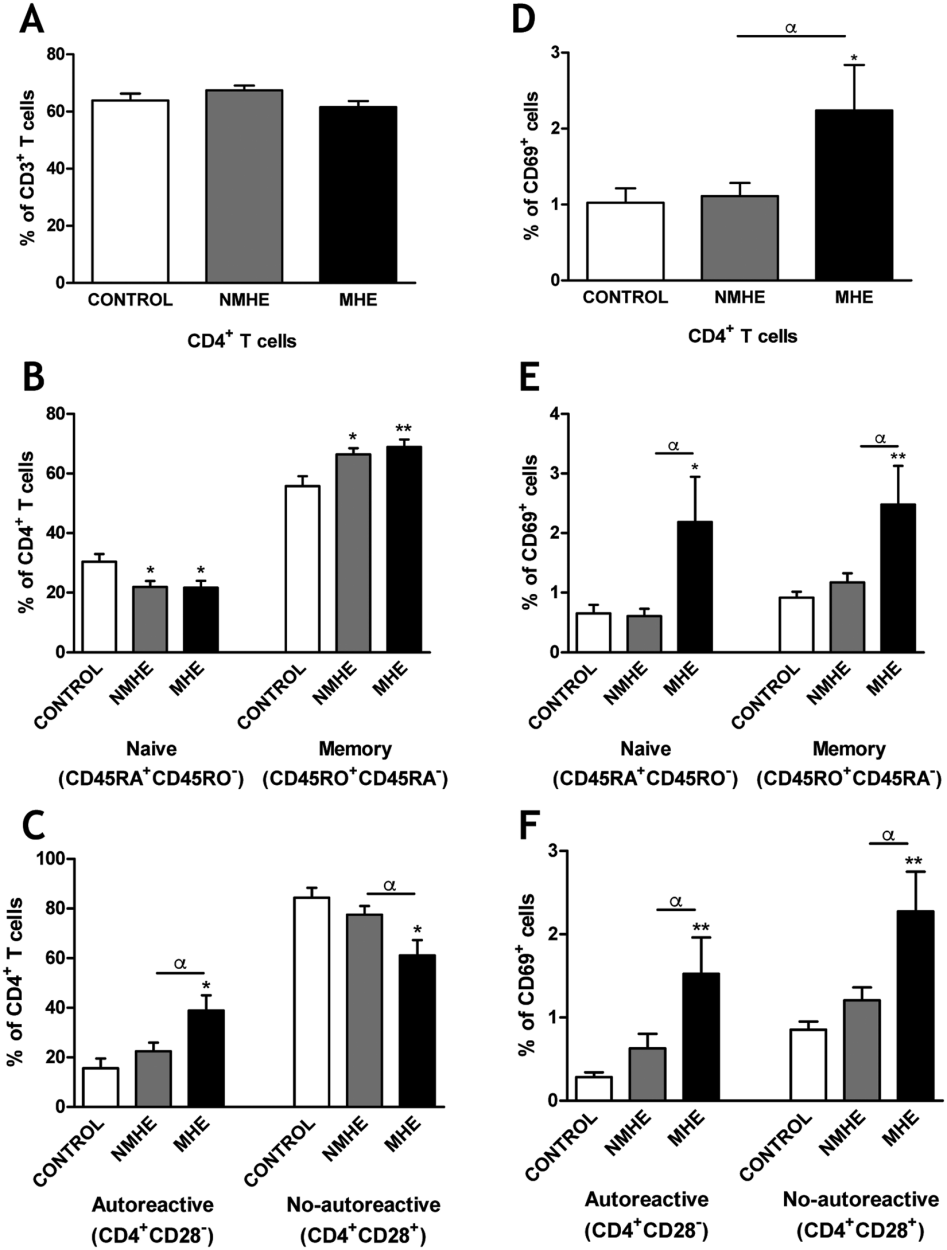
MHE is associated with expansion of autoreactive CD4^+^CD28^−^ T lymphocytes and induction of the early activation marker CD69 in CD4^+^ T lymphocytes in peripheral blood. (**A**) Percentage of total lymphocytes that are CD4^+^ T lymphocytes. (**B**) Percentage of CD4^+^ T lymphocytes that are naive or memory. (**C**) Percentage of CD4^+^ T lymphocytes that are autoreactive (CD4^+^CD28^−^) or not autoreactive (CD4^+^CD28^+^). (**D–F**) show that percentage of total CD4^+^ T lymphocytes (**D**), naive or memory CD4^+^ T lymphocytes (**E**), and autoreactive or not autoreactive CD4^+^ T lymphocytes (**F**) that express CD69. Values are the mean ± SEM of the following number of individuals: CONTROL n = 23; without MHE (NMHE) n = 30 and with MHE (MHE) n = 28. Values significantly different from controls are indicated by asterisks*. Values significantly different in patients with MHE compared to NMHE are indicated by α. (*/α p < 0.05; **p < 0.01).

Of the total (CD3^+^) T lymphocytes, the proportion of CD4^+^ lymphocytes was 64 ± 2% in controls and was not affected in patients without (67 ± 2%) or with (62 ± 2%) MHE (Fig. 2A).

Patients without MHE show more memory CD4^+^ T lymphocytes (66 ± 2%, p < 0.05) than control subjects (56 ± 3%). The increase was larger and more significant (69 ± 2%, p < 0.01) in patients with MHE (Fig. 2B). The percentage of naive CD4^+^ T lymphocytes was reduced in parallel in the patients (Fig. 2B), indicating a shift from naive to memory cells.

Most CD4^+^ lymphocytes are also CD28^+^ and require exposure to CD28 to be activated. Some CD4^+^ lymphocytes lack CD28 (CD4^+^CD28^−^) and are considered autoreactive^24–26^. The proportion of CD4^+^ lymphocytes that are CD4^+^CD28^−^ was expanded selectively in patients with MHE to 39 ± 6% (p < 0.05) compared to controls (15 ± 4%) or patients without MHE (22 ± 4%). The percentage of CD4^+^CD28^+^ T lymphocytes was reduced (p < 0.05) in parallel in patients with MHE (Fig. 2C).

We then assessed whether MHE is associated with increased CD4^+^ T lymphocytes activation. The expression of the early activation marker CD69 was specifically increased in CD4^+^ T lymphocytes from patients with MHE, but not from patients without MHE (Fig. 2D–F). The increase was general for all CD4^+^ T lymphocytes (2.2-fold, p < 0.05), for CD4^+^CD28^−^ (5.4-fold, p < 0.01); CD4^+^CD28^+^ (2.6-fold, p < 0.01); naive (3.3-fold, p < 0.05) and memory (2.7-fold, p < 0.01). This indicates a general activation of CD4^+^ T lymphocytes, especially of CD4^+^CD28^−^ in patients with but not without MHE (Fig. 2D–F).

**Figure 3:**
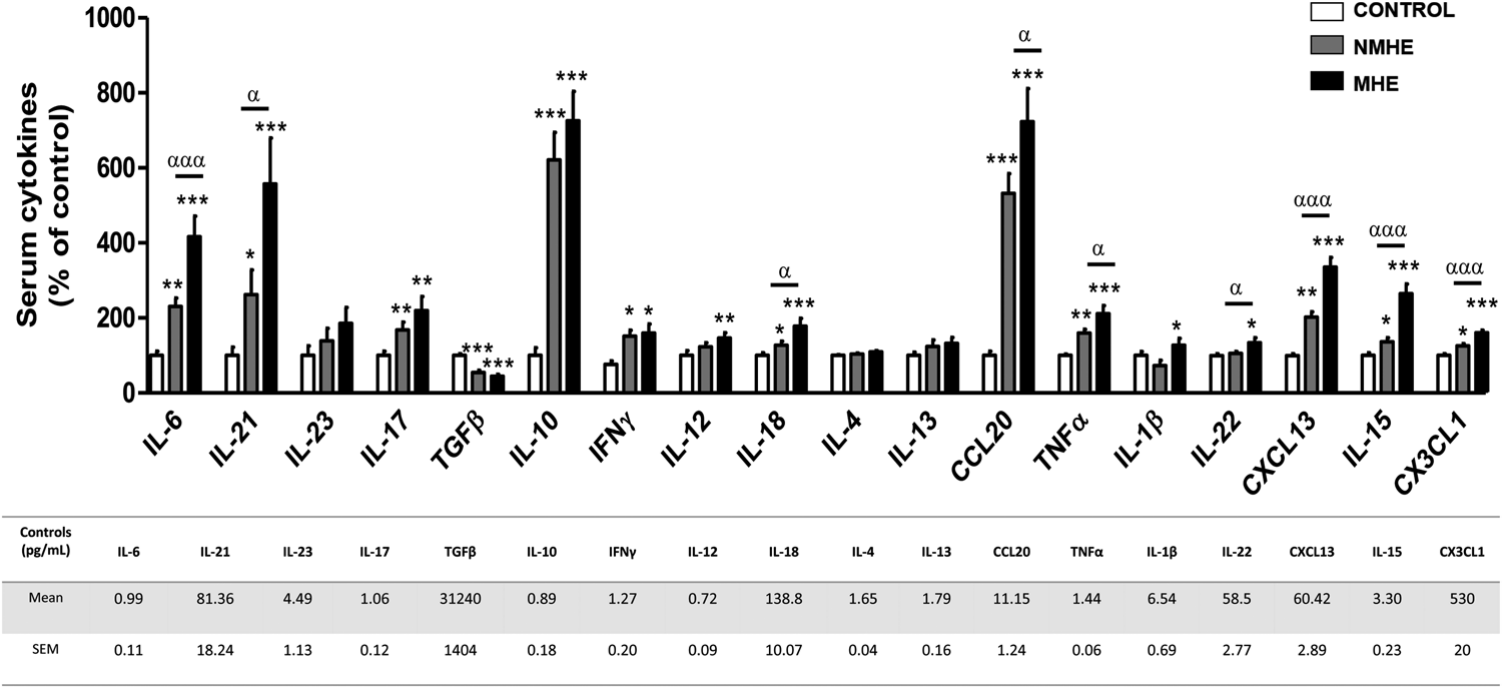
Serum levels of different cytokines. The serum levels of the indicated cytokines were measured and are expressed as percentage of controls to allow easy identification of alterations in patients without (NMHE) and with (MHE). The concentrations for controls are given in pg/ml in the Table. Values are the mean ± SEM of the following number of individuals: CONTROL n = 40; NMHE n = 50; MHE n = 40. Values significantly different from controls are indicated by asterisks*. Values significantly different in patients with MHE compared to NMHE are indicated by α. (*/^α^p < 0.05; **p < 0.01; ***/^ααα^p < 0.001).

We also analyzed a wide pattern of cytokines in serum (Fig. 3). In patients without MHE the levels of IL-6, IL-21, IL-17, IL-10, IFNγ, IL18, CCL20, TNFα, CXCL13, IL-15 and CX3CL1 (fractalkine) were increased and TGFβ was decreased compared to controls (Fig. 3). Patients with MHE show a more potent immunological response. The increases in IL-6, IL-21, IL-17, IL-10, IL-18, CCL20, TNFα, CXCL13, IL-15 and CX3CL1 (fractalkine) were higher than in patients without MHE. In addition the levels of IL-12, IL-1β and IL-22 were increased in patients with MHE but not in patients without MHE (Fig. 3).

**Figure 4:**
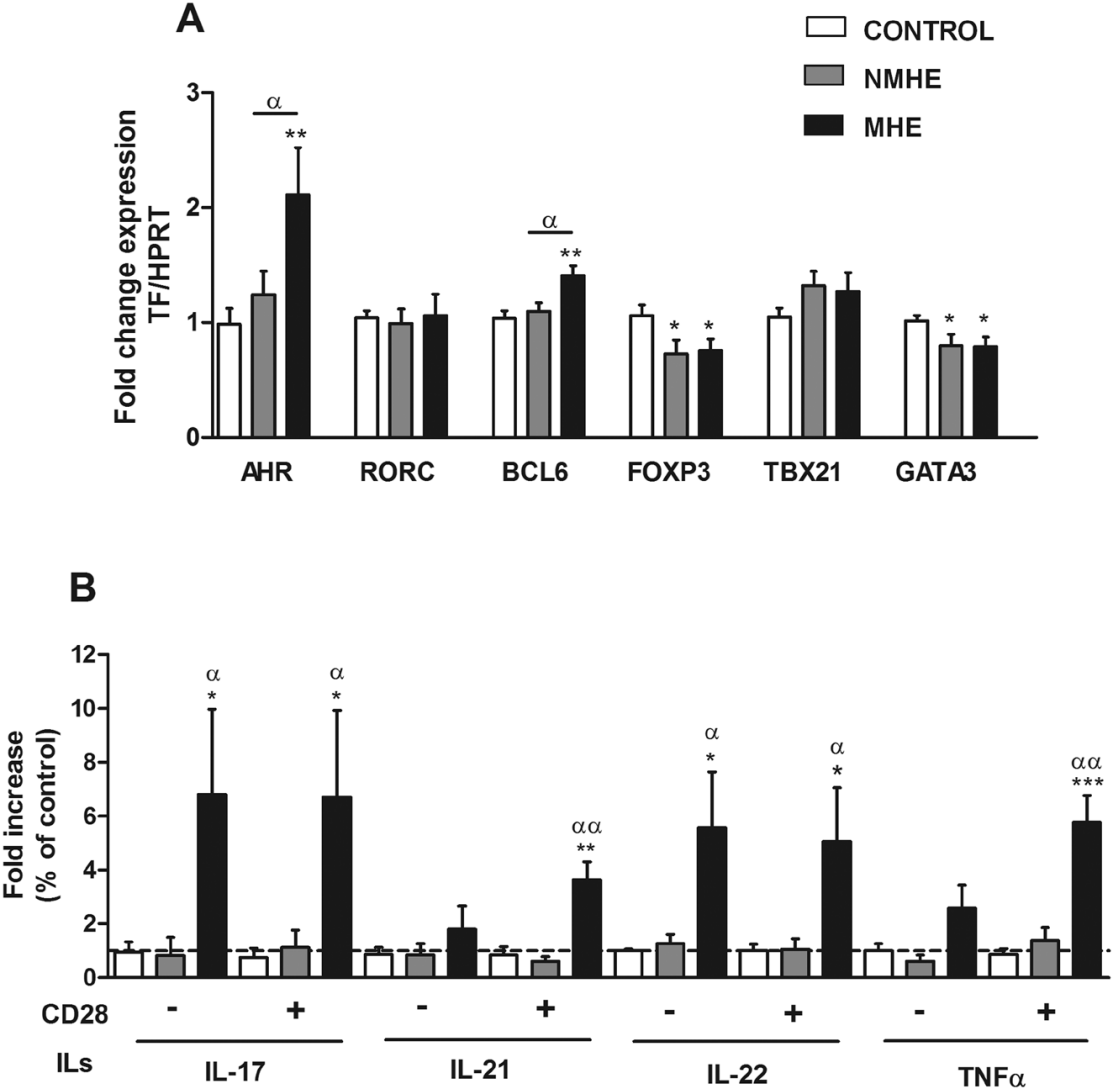
MHE is associated with expansion of Th follicular and Th22 CD4^+^ T lymphocytes subsets. (**A**) Analysis of the expression of the transcription factors AHR, RORC, BCL6, FOXP3, TBX21 and GATA3 mRNA expression in PBMCs. Data represent the normalized target gene amount relative to controls which are considered as 1. Values are the mean ± SEM of the following number of individuals: CONTROL n = 19; without MHE (NMHE) n = 15; with MHE (MHE) n = 15. (**B**) Analysis of cytokines secreted by isolated CD4^+^ T cells *in vitro* in the absence (−) or the presence (+) of added CD28. Values are given as the fold increase of cytokines levels over controls, which are considered as 1. Values are the mean ± SEM of the following number of individuals: CONTROL n = 9; NMHE n = 5; MHE n = 7. Values significantly different from controls are indicated by asterisks*. Values significantly different in patients with MHE compared to NMHE are indicated by α. (*/^α^p < 0.05; **/^αα^p < 0.01; ***p < 0.001).

CD4^+^ T lymphocytes may differentiate into different subsets characterized by the expression of specific transcription factors and by the production of certain cytokines. The expression of the transcription factors BCL6, specific for Tfh lymphocytes, and AHR, specific for Th22 lymphocytes were selectively increased (p < 0.01) to 140 ± 8% and 211 ± 39%, respectively, in patients with MHE but not without MHE (Fig. 4A). The transcription factors FOXP3 and GATA3, markers of iTreg and Th2 lymphocytes were decreased (p < 0.05) in patients with or without MHE compared to controls (Fig. 4A). No differences were found for RORC and TBX21, markers of Th17 and Th1 (Fig. 4A).

To better characterize the subsets of CD4^+^ T lymphocytes present in the patients, we incubated isolated CD4^+^ T lymphocytes in the absence or presence of added CD28 and measured the cytokines released to the supernatant. No change was observed in the cytokines released from patients without MHE compared to controls. In contrast, CD4^+^ T lymphocytes isolated from patients with MHE show a strong increase in the release of IL-17, IL-21, IL-22 and TNFα (Fig. 4B). For IL-21 and TNFα there was already a tendency to increase in the absence of added CD28 but the increase was only significant when CD28 is added. For IL-17 and IL-22 the increase was already significant in the absence of added CD28 (Fig. 4B), suggesting increased activation of Tfh, Th17 and Th22 cells.

**Figure 5:**
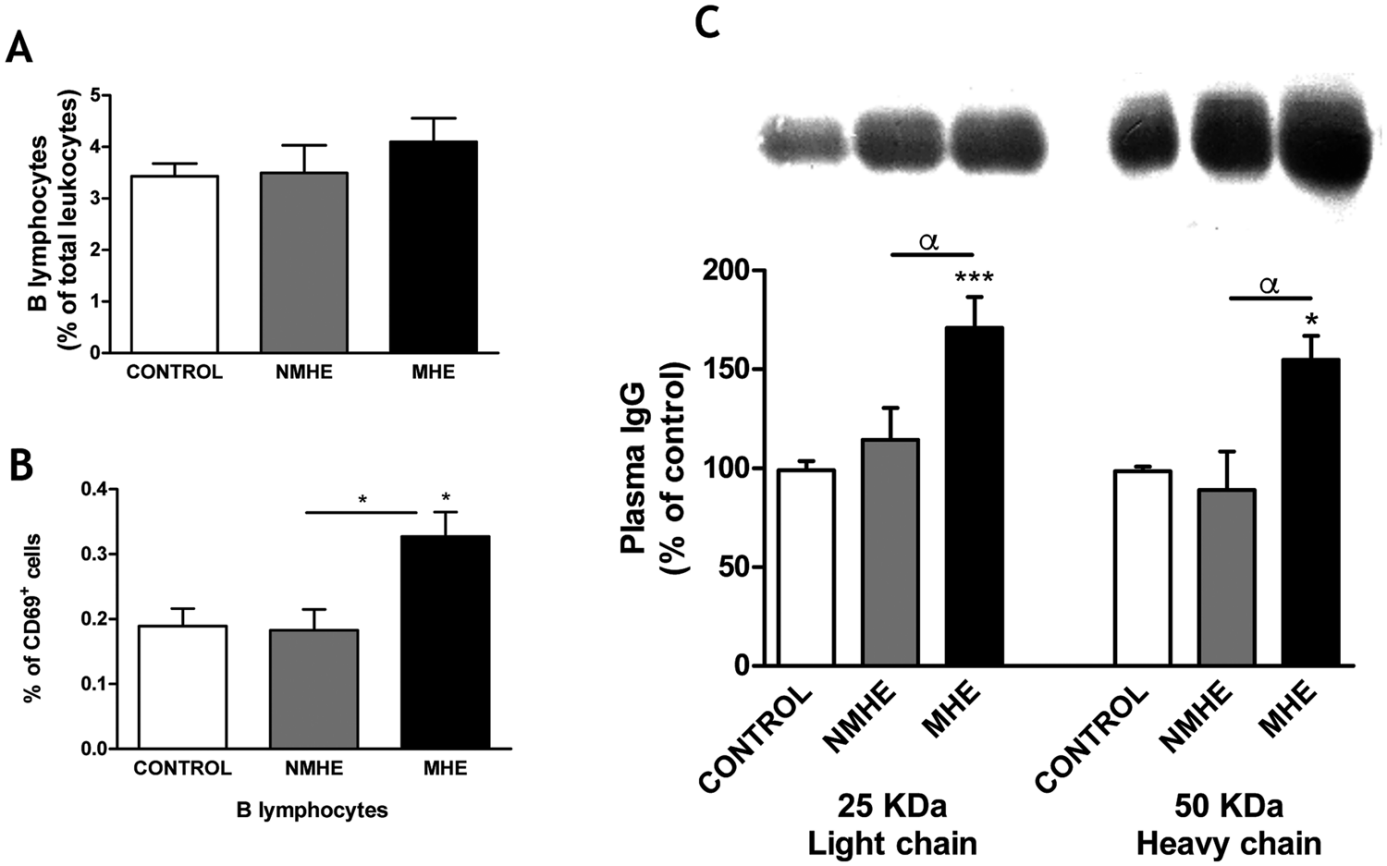
MHE is associated with activation of B lymphocytes and increased IgG content in plasma. (**A**) Percentage of total leukocytes that are B lymphocytes, as identified by flow cytometry by morphology combined with CD45 and CD19 positively marked cells. (**B**) Percentage of B lymphocytes that are activated (CD69^+^). (**C**) Content of IgG light (25 KDa) and heavy (50 KDa) chains in plasma, analyzed by western blot and expressed as percentage of control subjects. Representative images of the blots are shown. Unprocessed original scan of the blot is shown in Supplementary Fig. 1. Values are the mean ± SEM of the following number of individuals: CONTROL n = 19, without MHE (NMHE) n = 17, with MHE (MHE) n = 22. Values significantly different from controls are indicated by asterisks*. Values significantly different in patients with MHE compared to NMHE are indicated by α. (*/^α^p < 0.05; ***p < 0.001).

We also assessed the alterations in B lymphocytes. The percentage of B lymphocytes respect to total leukocytes was similar in controls and patients (Fig. 5A). B lymphocytes were selectively activated in patients with MHE but not in patients without MHE, as reflected by the increased proportion of B lymphocytes showing CD69 expression: 0.18 ± 0.03% in controls or patients without MHE and 0.33 ± 0.03% (p < 0.05) in patients with MHE (Fig. 5B). The increased activation of B lymphocytes is reflected in increased IgG content in plasma of patients with MHE (Fig. 5C). The contents of the 25 KDa (light) and 50 KDa (heavy) chains of IgG, were increased to 171 ± 15% (p < 0.001) and 155 ± 12% (p < 0.05) of controls, respectively (Fig. 5C).

The above data identify a series of immunological parameters which are specifically altered in patients with MHE. As shown in Table 1, there was a significant correlation between most of these parameters and cognitive impairment as reflected in the PHES.

**Table 1:** Diagnostic accuracy of different variables for detection of MHE.

Parameters	Correlation with PHES score^§^		Receiver operating characteristic (ROC) curves^§§^				
	Correlation coefficient	*p* value	AUROC (95% CI)	*p* value	Cutoff	Sensitivity (%)	Specificity (%)
IgG 25Kda	−0.521	0.002	0.78 (0.60–0.97)	0.018	124^a^	91	58
IL-15	−0.341	0.011	0.77 (0.64–0.90)	<0.001	5.682^b^	73	73
CXCL13	−0.541	<0.001	0.76 (0.63–0.88)	<0.001	159.6^b^	68	81
IL-6	−0.347	<0.001	0.75 (0.66–0.84)	<0.001	2.7^b^	70	76
CX3CL1	−0.517	<0.001	0,75 (0,62–0,88)	0.001	0,77^c^	70	69
%CD69 + (CD4 + CD28−)	−0.284	0.041	0.73 (0.57–0.88)	0.016	0.7^d^	50	72
%CD4 + CD28− (CD4)	−0.355	0.008	0.70 (0.54–0.86)	0.020	28.9^d^	63	72
IL-18	−0.218	0.009	0.68 (0.57–0.79)	0.003	199.8^b^	71	57
TNF-α	−0.420	0.001	0.67 (0.51–0.83)	0.052	—	—	—
IL-13	−0.254	0.030	0.61 (0.46–0.75)	0.153	—	—	—
CCL20	−0.319	<0.001	0.59 (0.48–0.71)	0.111	—	—	—
TGFb	0.459	<0.001	0.59 (0.42–0.75)	0.306	—	—	—
IL-10	−0.394	<0.001	0.58 (0.46–0.70)	0.197	—	—	—
IL-21	−0.186	0.038	0.58 (0.46–0.70)	0.197	—	—	—
%CD69 + (B lymphocytes)	−0.350	0.029	0.50 (0.29–0.71)	0.985	—	—	—

^§^Spearman’s Rho correlation between parameters and PHES score. ^§§^Receiver operating characteristic (ROC) curves for sensitivity and specificity of parameters in the diagnosis of minimal hepatic encephalopathy in the cohort of patients. AUROC, area under the receiver operating curve; CI, confidence interval; IL, interleukin; Cutoff: ^a^ in % of control, ^b^ in pg/mL, ^c^ in ng/mL and ^d^ in % over cell population.

We also analyzed the possible diagnostic utility of these parameters to detect the presence of MHE by performing a ROC analysis. As shown in Table 1, some of these parameters show diagnostic utility, especially the levels of the 25 KDa IgG subunit, IL-15, CXCL13, IL-6, CX3CL1, CD69^+^CD4^+^CD28^−^ and CD4^+^CD28^−^, which show areas under the curve of 0.78; 0.77; 0.76; 0.75; 0.75; 0.73 and 0.70, respectively (Table 1).

We also performed logistic regression analysis to assess which CD4^+^ T cell population contributes to cognitive impairment. On univariate analysis, MHE was associated with the percentages of CD4^+^CD28^−^ population, of CD69^+^ cells over the CD4^+^ naive cells and of CD69^+^ cells over the CD4^+^CD28^−^ population (Supplementary Table 2). Multivariate logistic regression analysis, using as dependent variable MHE and as independent variables CD4^+^T cell subsets, showed that the percentages of CD4^+^CD28^−^ population and of CD69^+^ cells over the same population were associated with cognitive impairment measured by PHES (Supplementary Table 2). When transaminases (ALT and AST), as indicator of liver damage, were included in the multivariate analysis, they were not predictive of MHE (Supplementary Table 2).

## Discussion

**Figure 6:**
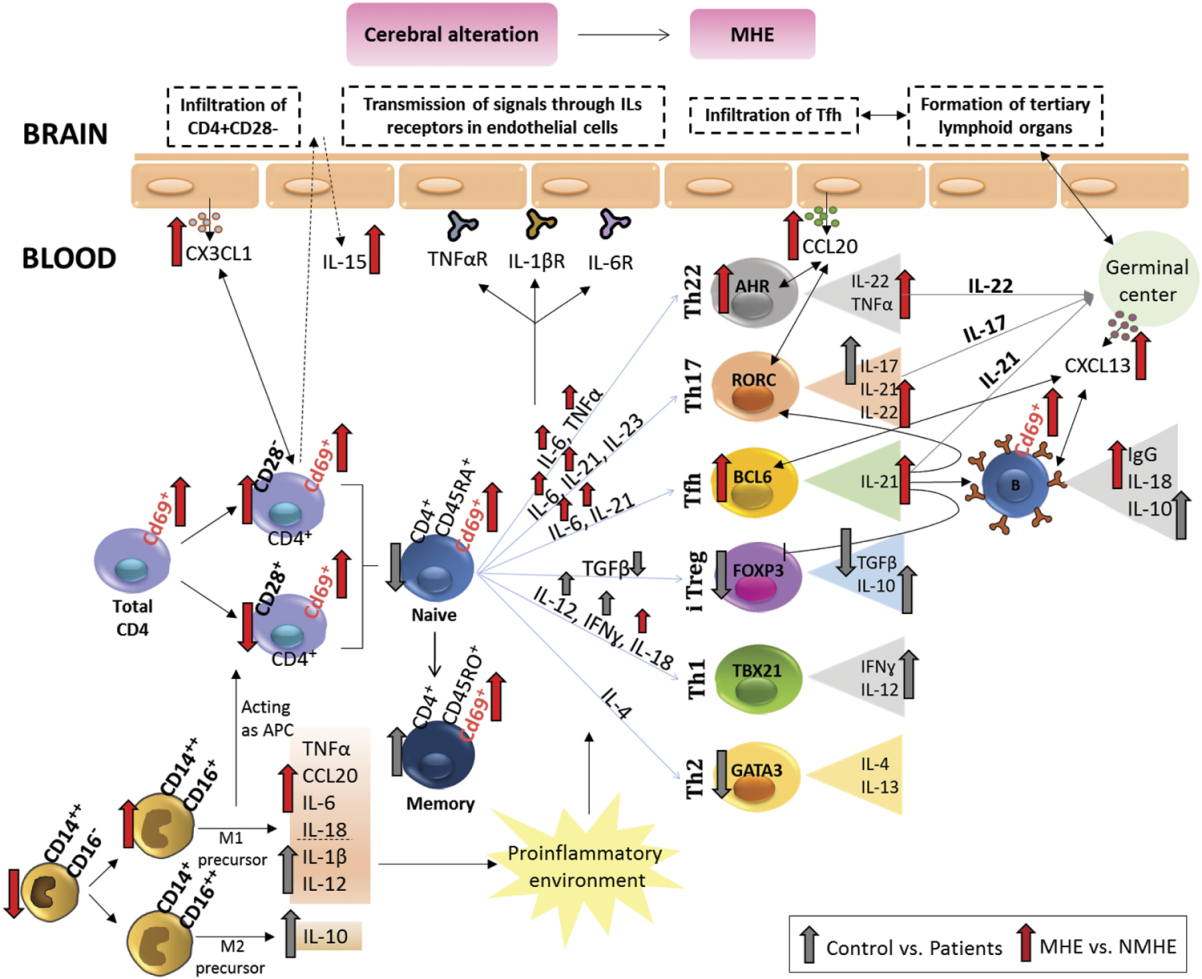
Scheme summarizing the changes in the immunophenotype associated with MHE and hypotheses on how this may affect the brain and lead to MHE. The main alterations associated with MHE are: (1) Increased activation of all subtypes of CD4^+^ T-lymphocytes, as indicated by the increased expression of CD69. (2) An increased amount of CD4^+^CD28^−^ T lymphocytes, associated with increased levels of CX3CL1 (fractalkine) and of IL-15, which may promote their infiltration into the brain. (3) Increased differentiation of CD4^+^ T lymphocytes to Th follicular and Th22, which may promote the formation of tertiary lymphoid organs in brain. (4) Increased activation of B lymphocytes and serum IgG. These four main alterations may contribute separately or jointly to alter cerebral function and to the appearance of the neurological alterations associated to MHE in cirrhotic patients. Some possible mechanisms by which changes in peripheral inflammation in patients with MHE may contribute to the appearance of the neurological alterations are: (**a**) infiltration into the brain of CD4^+^CD28^−^ T lymphocytes, leading to neuroinflammation and neurological impairment; (**b**) activation by peripheral interleukins (TNFα, IL-1b, IL-6) of their receptors in endothelial cells, triggering the release of inflammatory factors into the brain, neuroinflammation and neurological alterations; (**c**) infiltration of Tfh cells and formation of tertiary lymphoid organs with B lymphocytes germinal centers, leading to neurological alterations.

The results reported indicate that appearance of MHE in patients with liver cirrhosis is associated with specific changes in the immune system, different from those present in patients without MHE, as summarized in Fig. 6. The differences in inflammation and in the immunophenotype in patients with and without MHE could be due to different causes. One possibility could be that patients with MHE had stronger liver alterations, resulting in increased or different inflammation. The data in Supplementary Table 1 show that indicators of liver damage (transaminases, MELD, Child Pugh) are not different in patients with and without MHE. The multivariate analysis (Supplementary Table 2) also shows that liver damage alone, as measured by transaminases levels, is not predictive of MHE. Although progression of liver disease would contribute to induction of MHE, these data indicate that changes in the immunophenotype induce MHE independently of the grade of liver damage.

Patients included in the study did not show fever or any clinical or biological sign of recent infection, suggesting that the changes in immunophenotype associated to appearance of MHE are not due to infections. Another possibility, which is discussed below is that changes in the immunophenotype could be due to induction of an autoimmune response superimposed to liver failure. Why some patients show this response and MHE and other patients do not remain unclear for the moment. It is possible that some patients are more susceptible due to genetic, dietary, environmental or other causes.

A main finding is that patients with MHE present an expansion of autoreactive CD4^+^CD28^−^ lymphocytes (2.6-fold) which does not occur in patients without MHE. CD4^+^CD28^−^ T lymphocytes expand in autoimmune diseases and with ageing and have aberrant functions which contribute to progression and maintenance of chronic immune diseases and to disease- and age-related immune dysfunctions^25, 26, 31^. In pathological states, CD4^+^CD28^−^ T cells represent prematurely senescent cells resulting from persistent immune activation and are considered a biomarker for early immunosenescence. The degree of accumulation of CD4^+^CD28^−^ T cells predicts the severity of clinical manifestations^32^. The increased amount of CD4^+^CD28^−^ T cells in MHE may be reflecting early immunosenescence and could be a biomarker of immunological dysfunction and of MHE.

CD4^+^CD28^−^ T lymphocytes arise through repeated antigenic stimulation in autoimmune and chronic inflammatory disorders and have a cytotoxic phenotype and inbuilt pro-inflammatory ability^33^. They produce large amounts of TNFα and IFNγ and promote a pro-inflammatory environment. CD4^+^CD28^−^ T cells express perforin and granzyme B and can directly lyse endothelial cells and damage tissues^25, 26, 31^. They also express CD161, a molecule that facilitates trans-endothelial migration and tissue invasion^32, 34^ and CX3CR1, the fractalkine receptor, which is not expressed by CD4^+^CD28^+^ T cells^35^. CD4^+^CD28^−^ T cells are also expanded and infiltrate brain tissue in patients with multiple sclerosis^24, 35, 36^. The infiltration of CD4^+^CD28^−^ T cells is driven by CX3CR1^24, 35, 36^ and amplified by IL-15, which also amplifies the pathogenic properties of CD4^+^CD28^−^ T cells^33^.

The increased amount of CD4^+^CD28^−^ T lymphocytes in patients with MHE indicates stronger immune dysfunctions than in patients without MHE, with persistent immune activation, increased production of TNFα, IFNγ and of molecules which may damage endothelial cells and tissues. This could facilitate their infiltration in brain, as occurs in multiple sclerosis. Patients with MHE also show increased CX3CL1 (fractalkine) levels which may activate CX3CR1 and of IL-15, which may promote infiltration into the brain of CD4^+^CD28^−^ T cells. This is one of the mechanisms which could contribute to appearance of neurological alterations in patients with MHE (Fig. 6).

Another change that occurs in patients with MHE but not in those without MHE is an increase in the early activation marker CD69 in all subsets of CD4^+^ T lymphocytes, especially in CD4^+^CD28^−^ T cells. CD69 is persistently expressed by infiltrating T cells in chronic inflammation^37–39^. CD69^+^CD4^+^ T cells are continuously activated by self-peptides and are pathological through abnormal regulatory effects on cytokine balance^40^. CD69 also controls T cell differentiation^41, 42^.

The increase in CD69 in patients with MHE would indicate a persistent activation of CD4^+^ T lymphocytes which may contribute to immune dysfunction, to altered patterns of cytokines and of CD4^+^ T cells differentiation and facilitate tissue infiltration.

Patients with MHE also show an abnormal pattern of differentiation of CD4^+^ T cells into T-helper subsets. Naive CD4^+^ T cells may differentiate into different effector T helper (Th) and regulatory (Treg) cell subsets: Th1, Th2, Th17, Th22, Th follicular (Tfh) or iTreg with specialized functions^27, 43, 44^. The main cytokines driving differentiation to each subset of CD4^+^ T cells, the transcription factors characterizing each subset and the main cytokines they release are indicated in Fig. 6. Dysregulation of this differentiation results in the pathogenesis of different autoimmune and inflammatory diseases^44^.

CD4^+^ T cells differentiation is mediated by the integration of signals provided by antigen presenting cells, interacting surface receptors and cytokines^44, 45^. The fate of CD4^+^ T cells differentiation is also controlled by the strength of the T cell receptor (TCR) activation. Strong TCR activation favors Th1, Th17 and Tfh cells differentiation, while weak TCR signal promotes Th2 and iTreg^44, 46^.

To characterize the Th and iTreg cell subsets present in patients with and without MHE we analyzed the key transcription factors, the cytokines produced by isolated CD4^+^ T cells and the pattern of serum cytokines.

Patients with MHE show increased differentiation into Th follicular (Tfh) cells, characterized by expression of the transcription factor BCL6 and release of IL-21^47, 48^. Patients with MHE, but not patients without MHE, show increased BCL6 expression and a strong increase in serum IL-21. Moreover, CD4^+^ T cells isolated from patients with MHE release IL-21 *in vitro*, while cells from patients without MHE did not. These data support that patients with MHE show increased differentiation of CD4^+^ T cells into Th follicular cells, which does not occur in patients without MHE.

As mentioned above, differentiation into Tfh cells requires strong TCR activation, which also favors Th17 differentiation and reduces differentiation into Th2 and iTreg cells. The expression of the transcription factors FOXP3 and GATA3, characteristic for iTreg and Th2 cells respectively is reduced in patients with or without MHE, suggesting that activation of TCR is stronger in patients than in controls and is stronger in patients with MHE (leading to Tfh differentiation) than without MHE.

Patients with MHE, but not those without MHE, also show increased differentiation into Th22 cells, characterized by expression of the transcription factor AHR and release of IL-22 and TNFα^49^. MHE patients show increased AHR expression and increased serum levels of IL-22. Moreover, their CD4^+^ T cells release IL-22 *in vitro*, while cells from patients without MHE did not. Th22 cells are implicated in the pathogenesis and in the neurological alterations in autoimmune diseases^50^. Th22 cells and IL-22 are increased in patients with multiple sclerosis. Treatment of these patients with IFNβ-1a suppresses Th22 and Th17 cell responses and decreases demyelination^51^. Increased Th22 would also contribute to the appearance of the neurological alterations in patients with MHE.

The increased differentiation to Tfh and Th22 cells in patients with MHE may contribute to formation of tertiary lymphoid organs (TLOs), activation of B lymphocytes, as supported by the increased CD69 expression, and increased antibodies formation, as supported by the increased IgG levels in patients with MHE.

During chronic immune responses effector cells can infiltrate target tissues and organize into B cell follicles containing germinal centers and T cell areas. This phenomenon is called formation of tertiary lymphoid organs (TLOs). TLOs have been identified in different tissues in different diseases, including the brain in multiple sclerosis^52^. IL-22 plays a main role in the formation of TLOs in autoimmune diseases. IL-22 promotes CXCL13 expression, pivotal for B cell recruitment and TLOs formation, which contribute to B-cell activation and infiltration and autoimmune pathogenesis^43^. Therapeutic blockade of IL-22 inhibits CXCL13 expression and reduces TLOs formation, B cells aggregation and autoantibodies formation^53, 54^.

The expansion of Th22 cells in patients with MHE could facilitate the infiltration of immune cells and formation of TLOs into the brain which may contribute to cognitive impairment, as occurs in patients with multiple sclerosis. As far as we know, infiltration of immune cells in brain of patients with MHE or HE has not been reported. Further studies to assess this possibility could help to better understand the pathogenesis of MHE and HE.

Infiltration of immune cells and formation of TLOs could be also facilitated in patients with MHE by the increased differentiation of CD4^+^ T cells into Tfh cells, the specialized providers of B cell help. Tfh cells are necessary for the formation and maintenance of germinal centers (GCs) and to regulate B cells differentiation into plasma cells and memory B cells. Tfh cell migrate in response to CXCL13 and relocate to follicles and can play important roles in autoimmune diseases^55^. Patients with MHE show, in addition to increased differentiation of CD4^+^ T cells into Tfh cells, a 3.3-fold increase in serum CXCL13. This suggests that more Tfh would migrate to germinal centers in patients with MHE, resulting in altered modulation of B cells by Tfh in MHE. In fact, patients with MHE show a strong increase in CD69 expression in B lymphocytes, indicating stronger activation, and also a strong increase of IgG in plasma.

The increased CCL20 levels in patients with MHE may also contribute to T and B lymphocytes infiltration. CCL20 (also known as MIP-3a) is a chemoattractant of memory T cells and B lymphocytes^56, 57^ and is strongly induced in endothelial cells by TNFα. CCL20 could therefore contribute to the infiltration of T and B lymphocytes^56, 57^ also in patients with MHE.

Activated B-lymphocytes infiltrate brain tissue in stroke and multiple sclerosis both in mice and humans and induce an autoimmune response that mediates cognitive impairment, which is prevented by ablation of B cells by genetic or pharmacological manipulations^58, 59^. T lymphocytes would be involved in the mechanisms by which B lymphocytes induce cognitive dysfunction. T lymphocytes alone seem to be not sufficient to induce cognitive deficits but the joint presence of T and B lymphocytes induces them^58^. There is a trafficking of B cells between peripheral blood and the brain in multiple sclerosis. B cell follicle like-structures have been found in the meninges of multiple sclerosis patients^60, 61^. B cells prime and regulate T cells and play a main role in multiple sclerosis pathogenesis, as clearly shown by the clinical success of B cell targeting therapies^60^. Increased activation of B lymphocytes and antibodies production could be therefore another mechanisms contributing to appearance of MHE in cirrhotic patients.

Another mechanism by which peripheral inflammation may induce neurological alterations in MHE is the activation by peripheral interleukins (TNFα, IL-1β, IL-6) of their receptors in endothelial cells, triggering the release of inflammatory factors into the brain, neuroinflammation and neurological alterations. For example, in rats injected with LPS, blood IL-6 activates its receptors in endothelial cells leading to activation of STAT3 which increases cyclooxygenase 2 and PGE2 in cerebral cortex^62^.

The results reported in the present work provide relevant new information on the mechanisms involved in the appearance of MHE. Understanding the pathophysiology of the appearance of MHE is important to be able to design new, more effective treatments to reverse MHE and the associated neurological alterations.

Moreover, the results reported also may have practical and clinical interest, allowing early detection of MHE. MHE is currently diagnosed mainly using psychometric tests. MHE is not diagnosed in many clinical settings due to lack of simple procedures and many patients remain undiagnosed and untreated. There is therefore an important practical and clinical interest in detecting MHE at early stages based on laboratory parameters to allow beginning treatment of the patients. Early treatment improves cognitive functions and health-related quality of life in patients with cirrhosis who have MHE. Lactulose improves cognitive functions and health-related quality of life in patients with cirrhosis who have MHE^63^. Sidhu *et al*.^64^ showed that rifaximin improves psychometric performance and health-related quality of life and reverse MHE in 75% of patients with MHE and lactulose in 69% of patients with MHE^65^. De Rui *et al*.^66^ recently reviewed the data on treatment of MHE and reported that from 43 papers accessible on PubMed from 1993 to 2015, all studies, except for one document that treatments are effective in patients with MHE to improve cognitive functions and health-related quality of life in patients with cirrhosis who have MHE.

In summary, this study has identified specific alterations of the immune system associated with appearance of the neurological alterations in cirrhotic patients with MHE. The main alterations associated with MHE are: 1) increased activation of all subtypes of CD4^+^ T lymphocytes, as indicated by increased CD69 expression; 2) increased amount of CD4^+^CD28^−^ T lymphocytes, associated with increased levels of CX3CL1 (fractalkine) and of IL-15, which may promote their infiltration into the brain; 3) increased differentiation of CD4^+^ T lymphocytes to Tfh and Th22, which may promote the formation of tertiary lymphoid organs; 4) increased activation of B lymphocytes and serum IgG. These four main alterations may contribute separately or jointly to the appearance of the neurological alterations associated to MHE in cirrhotic patients.

As summarized in Fig. 6, these results suggest a few possible mechanisms by which changes in peripheral inflammation in patients with MHE may contribute to the appearance of the neurological alterations: a) infiltration into the brain of CD4^+^CD28^−^ T lymphocytes, leading to neuroinflammation and neurological impairment; b) activation by peripheral interleukins (TNFα, IL-1β, IL-6) of their receptors in endothelial cells, triggering the release of inflammatory factors into the brain, neuroinflammation and neurological alterations; c) infiltration of Tfh cells and formation of tertiary lymphoid organs with B lymphocytes germinal centers, leading to neurological alterations.

Further studies to better characterize the contribution of these immunological changes to the neurological alterations and their possible utility in early diagnosis of MHE in cirrhotic patients are worth to be carried out.

## Materials and Methods

### Patients and controls

187 patients with liver cirrhosis were consecutively recruited from the outpatient clinics in the Hospitals Clínico and Arnau de Vilanova of Valencia, Spain. The diagnosis of cirrhosis was based on clinical, biochemical and ultrasonographic data. Exclusion criteria were: overt hepatic encephalopathy, recent (<6 months) alcohol intake, infection, recent (<6 weeks) antibiotic use or gastrointestinal bleeding, recent (<6 weeks) use of drugs affecting cognitive function, presence of hepatocellular carcinoma, or neurological or psychiatric disorder. Patients included in the study did not show fever or any clinical or biological sign of recent infection. None of the patients included in the study had hypothyroidism or altered TSH. 98 healthy volunteers were also enrolled in the study once liver disease was discarded by clinical, analytical, and serological tests. Blood ammonia was measured immediately after blood collection with the Ammonia Test Kit II for the PocketChemBA system (Arkay, Inc., Kyoto, Japan) the same day of psychometric test performance. All participants were included in the study after signing a written informed consent. Study protocols were approved by the Scientific and Ethical Committees of both hospitals. The procedures followed were in accordance with the ethical guidelines of the Declaration of Helsinki.

#### Diagnosis of MHE

MHE was diagnosed using the Psychometric Hepatic Encephalopathy Score (PHES) which comprises 5 psychometric tests^67, 68^. The scores were adjusted for age and education level using Spanish normality tables (www.redeh.org). Patients were classified as MHE when the score was ≤−4 points.

### Characterization of leukocyte population in whole blood by flow cytometry

Venous blood samples were taken in BD Vacutainer^®^ (Becton, Dickinson and Company, Franklin Lakes, NJ, USA) tubes with EDTA. 50 μL of whole blood was incubated with a mixture of monoclonal antibodies specific for the different leukocyte subpopulations (see below) and with 2 mL BD FACS Lysing Solution 1X (Becton, Dickinson and Company, Franklin Lakes, NJ, USA). Samples were incubated in the dark for 10 minutes at room temperature. Then, 50 µl of Flow Count (Beckman Coulter, Miami, FL, USA) was added in order to quantify the number of cells per microliter.

Analysis was performed on a Gallios flow cytometer (Beckman Coulter, Miami, FL, USA) and the Kaluza software package was used to analyze the flow cytometry data. The cytometer settings were performed as in ref. 69.

#### Monoclonal antibodies used

Different cell populations were labeled with antibodies to CD45 (total leukocytes), CD14 and CD16 (monocytes), CD3 (T lymphocytes), CD4 (T helper lymphocytes), CD19 (B lymphocytes), CD28 (negative selection for autoreactive T helper lymphocytes), CD69 (activated lymphocytes) and CD45RA and CD45RO (naive and memory T lymphocytes).

The antibodies used were the following: CD45-Krome Orange (clone J.33) (CD45-KO), CD4-PhycoerythrinTexas Red-X (Clone SFCI12T4D11 (T4)) (CD4- ECD), CD19- Allophycocyanin-Alexa Fluor 700 (clone J3.119) (CD19-APC-AlexaFluor700) and CD16-Allophycocyanin -Alexa Fluor 750 (clone 3G8) (CD16-APC-AlexaFluor750) from Beckman Coulter (Miami, FL, USA) and CD14-Pacific Blue (clone M5E2) (CD14-PB), CD3- Allophycocyanin (clone UCHT1) (CD3-APC), CD28-Pacific Blue (clone CD28.2) (CD28-PB), CD69- Phycoerythrin (clone FN50) (CD69-PE), CD45RA- Peridinin Chlorophyll Protein/Cyanin5.5 (clone HI100) (CD45RA-PERCP/Cy5.5) and CD45RO-Allophycocyanin/Cyanin7 (clone UCHL1) (CD45RO- APC/Cy7) from Biolegend (San Diego, CA, USA).

#### Determination of cytokine levels in serum

Blood samples were kept at room temperature for 30 minutes and then centrifuged for 10 minutes at 1.500 g. Following centrifugation, the serum or plasma samples were immediately separated and kept at −80 °C for subsequent cytokine analysis. Concentration of IL-6, IL-18, IL-17, IL-23, IL-21, IL-13, IL-4, TGFβ, IL-22, IL-1β (Affymetrix eBioscience, Vienna, Austria), IL-10, IL-15, INFγ, IL-12, CCL20, TNFα, CXCL13 and CX3CL1 (R&D Systems, Minneapolis, MN, USA) were measure by ELISA according to the manufacturer’s instructions. High sensitivity kits were necessary for IL-17, IL-4 and TNFα assessment.

### Analysis of transcription factors by quantitative PCR

RNA was extracted from peripheral blood mononuclear cells (PBMCs) with an RNAspin Mini RNA Isolation Kit according to the manufacturer’s directions (GE Healthcare, Buckinghamshire, UK). The quality of RNA was checked by spectrophotometry and samples with a ratio of 2.0 for absorbance at 260 nm relative to that at 280 were selected to generate cDNA. RNA was retro-transcript into cDNA in one step with the High-Capacity RNA-to-cDNA Kit according to the manufacturer’s instructions. Taqman^®^ assays labeled with FAM dye (see below) and the Gene Expression Master Mix were used for the real-time PCR (40 cycles) (all reagents were from Applied Biosystems, Foster City, CA, USA).

#### Taqman^®^ gene expression assays

TBX21 (Hs00203436_m1), GATA3 (Hs00231122_m1), BCL6 (Hs00153368_m1), RORC (Hs01076122_m1), FOXP3 (Hs01085834_m1) and AHR (Hs00907314_m1). ΔΔCt method was used to determine targets expression using HPRT1 (Hs02800695_m1) as a normalizer.

### Analysis of cytokines released from isolated CD4^+^ cells

CD4^+^T lymphocytes were isolated according with manufacturer’s directions using the CD4^+^T Cells Isolation Kit Human (Miltenyi Biotec, Germany). Isolated CD4^+^ cells were incubated with CD3 and with or without CD28 antibodies (purified mouse anti-human CD3 and CD28, respectively) from BD (Becton, Dickinson, Franklin Lakes, USA). In the presence of co-stimulation, a T cell become committed to activation after 6 hours of T cell receptor triggering; in the absence of co-stimulation, 30 hours are required^70, 71^. After 6 hours at 37 °C with 5% CO_2_, supernatant and cell pellets were collected for further analysis. IL-17, IL-22 and TNFα were measured in the supernatant using Elisa kits (see above) and IL-21 in the cell pellet by western blot.

### Determination of IgG level in plasma by western blot

Total protein of serum samples was quantified by a standard bicinchoninic acid assay (BCA Protein Assay Kit, Thermo Scientific, Rockford, IL, USA). 80 μg of total protein was heated 5 min at 100 °C with sample buffer 2X (Tris-HCl 0.5 M pH 6.8, glycerol 5%, SDS 10%, 2-mercaptoethanol 2.5%, bromophenol 1%) and then separated by 10% SDS-PAGE and transferred to nitrocellulose. Samples containing 80 μg of total plasma protein were subjected to electrophoresis and immunoblotting as in ref. 72 using anti-human IgG−Alkaline phosphatase mouse monoclonal antibody (clone GG-5, 1:80.000) (Sigma-Aldrich, St. Louis, MO, USA). The images were captured using the ScanJet 5300 C (Hewlett- Packard, Amsterdam, the Netherlands) and band intensities quantified using the Alpha Imager 2200, version 3.1.2 (AlphaInnotech Corporation, San Francisco).

### Statistical analysis

Values are given as mean ± standard error (SEM). Results were analyzed by one-way analysis of variance (ANOVA) followed by post-hoc Tukey test. Bivariate correlations were evaluated by Spearman correlation test. Univariate and multivariate logistic regressions were performed using the presence of MHE as dependent variable. Diagnostic utility was evaluated using receiver Operating Characteristic (ROC) curves. The significance level was set at P < 0.05.

### Data availability statement

All data generated or analysed during this study are included in this published article (and its Supplementary Information file).

## Electronic supplementary material

Supplementary Information
