# Supplementary material for: Minimal hepatic encephalopathy is associated with expansion and activation of CD4+CD28−, Th22 and Tfh and B lymphocytes
Source: Sci Rep. 2017 Jul 27;7:6683. doi: 10.1038/s41598-017-05938-1 (PMC5532287; doi:10.1038/s41598-017-05938-1)
Supplement: Supplementary file 1 — Supplementary Information [file 41598_2017_5938_MOESM1_ESM.doc]

**SUPPLEMENTARY MATERIAL**

**Minimal hepatic encephalopathy is associated with expansion and activation of**

**CD4^+^CD28^-^, Th22 and Tfh and B lymphocytes**

Alba Mangas-Losada, Raquel García-García, Amparo Urios, Desamparados Escudero-García, Joan Tosca, Remedios Giner-Durán, Miguel Angel Serra, Carmina Montoliu^*^, Vicente Felipo

**Contents:**

- **Supplementary Figure 1.** Unprocessed original scans of the blots from Figure 5
- **Supplementary Table 1.** Composition of the different groups and etiology of liver disease
- **Supplementary Table 2**. Univariate and multivariate logistic regression analysis of different CD4^+^ T cell population and indices of liver failure to predict MHE

**Supplementary Figure 1.** Unprocessed original scans of the blots from Figure 5


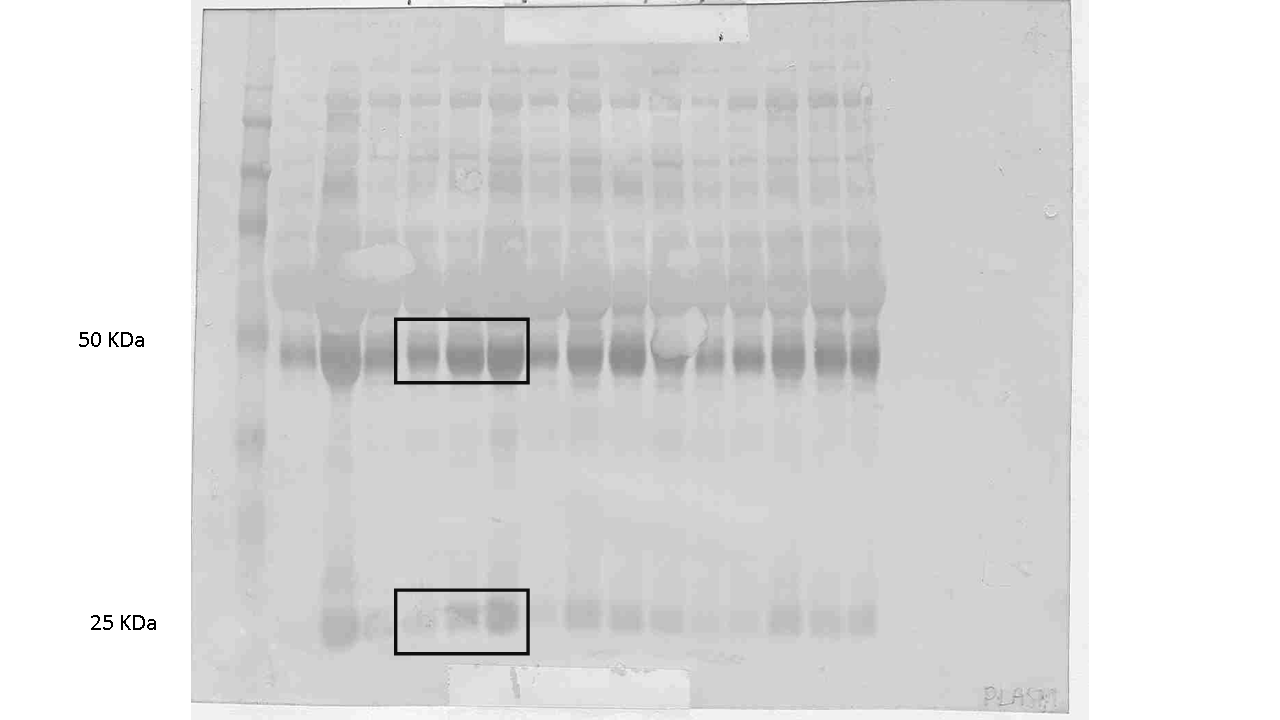


|  | **CONTROL PATIENTS** | | |
| --- | --- | --- | --- |
|  |  | **NMHE** | **MHE** |
| **Total individuals** | 98 | 125 | 62 |
| **Gender (M/F)** | 51/47 | 99/26 | 43/19 |
| **Age** | 57 ± 1 | 60 ± 1 | 64 ± 1 |
| **Alcohol** | ----- | 56 | 25 |
| **HBV/ HCV** | ----- | 51 | 22 |
| **Alcohol +HBV/ HCV** | ----- | 4 | 6 |
| **Others** |  | 14 | 9 |
| **Child Pugh A/B/C** | ----- | 103/22/0 | 41/17/4 |
| **MELD** | ----- | 9 ± 0.3 | 10 ± 0.4 |
| **Haemoglobin (g/dL )** | 14 ± 0.2 | 13 ± 0.2** | 12.5 ± 0.3*** |
| **Total bilirubin (mg/dL)** | 0.58 ± 0.07 | 1.21 ± 0.11** | 1.21 ± 0.14* |
| **ALT (U/L)** | 19.3 ± 1.6 | 35 ± 6.5* | 41 ± 7* |
| **AST (U/L)** | 21 ± 1.74 | 49.6 ± 9* | 61 ± 9* |
| **Alkaline phosphatase (mU/mL)** | 98 ± 18 | 242 ± 28** | 259 ± 27** |
| **GGT (U/L)** | 24.4 ± 3 | 156 ± 35* | 145 ± 34* |
| **CRP (mg/L)** | 2.1 ± 0.35 | 4.0 ± 0.8* | 4.0 ± 0.9* |
| **Sodium (mM )** | 140 ± 0.5 | 140 ± 0.5 | 139 ± 0.7 |
| **Ammonia (μM)** | 11 ± 0.5 | 26 ± 2*** | 33 ± 3*** |
| **TSH (μU/mL)** | 2 ± 0.3 | 2.6 ± 0.3 | 3 ± 0.4 |

**Supplementary Table 1.** Composition of the different groups and etiology of liver disease. Values are expressed as mean ± SEM. Abbreviations: NMHE, no minimal hepatic encephalopathy; MHE, minimal hepatic encephalopathy; M, male; F, female; HBV, hepatitis B virus; HCV, hepatitis C virus; MELD, model end stage liver disease. The Child Pugh Score is derived from a score of 1–3 given for severity of ascites, hepatic encephalopathy, INR, albumin and bilirubin. The higher the score is, the more severe the liver disease; ALT, alanine aminotransferase; AST, aspartate aminotransferase; GGT, gamma-glutamyl transpeptidase; CPR, C-reactive protein; TSH, thyroid-stimulating hormone. Asterisk (*) represents a significant difference with control. There are no differences between patients without and with MHE in any parameter (* p<0.05; ** p<0.01).

|  | OR | 95% CI | *p* value |
| --- | --- | --- | --- |
| Univariate logistic regression analysis | |  |  |
| Independent variables |  |  |  |
| CD4^+^ T cell population |  |  |  |
| %CD4 naive | 1.007 | 0.930-1.090 | 0.867 |
| %CD4 memory | 0.994 | 0.938-1.053 | 0.836 |
| **%CD4^+^CD28^-^** | 1.037 | 1.009-1.066 | **0.010** |
| **%CD69^+^(CD4 naive)** | 1.598 | 1.003-2.545 | **0.048** |
| %CD69^+^(CD4 memory) | 1.061 | 0.856-1.314 | 0.591 |
| **%CD69^+^(CD4^+^CD28^-^)** | 1.949 | 1.068-3.554 | **0.030** |
| Indices of liver damage |  |  |  |
| ALT (U/L) | 1.002 | 0.995-1.009 | 0.567 |
| **AST (U/L)** | 1.011 | 1.000-1.022 | **0.043** |
|  |  |  |  |
| Multivariate logistic regression analysis | | |  |
| Predictor variables |  |  |  |
| **%CD4^+^CD28^-^** | 1.058 | 1.016-1.101 | **0.006** |
| **%CD69^+^(CD4^+^CD28^-^)** | 2.675 | 1.256-5.695 | **0.011** |

**Supplementary Table 2**. Univariate and multivariate logistic regression analysis of different CD4^+^ T cell population and indices of liver failure to predict MHE.

On both uni- and multivariate analyses, the dependent variable was the presence of MHE according to PHES. On multivariate analysis, independent variables were those that were significant (p<0.05) on univariate analysis. OR, Odds ratio; CI, Confidence Interval; ALT, alanine aminotransferase; AST, aspartate aminotransferase.
